# Shortsightedness

**Published:** 1884-04

**Authors:** 


### SHORTSIGHTEDNESS.

MYOPIA or shortsightedness does not appear among the untutored savages; it is prevalent in civilized countries, but especially so in Germany, where the subject of bettering the eyesight of the nation is receiving a great amount of attention. Among the causes of myopia have been found the overtaxing of the strength of children, as well as their eyes, the use of books poorly printed on rough paper, with small type, reading and writing by a dim light, the use in school-rooms of desks not inclined nor of the proper height to enable each pupil to sit upright when writing, together with any work requiring the close application of the eyes for a considerable time. It has also been shown by some authorities that the German type is much more likely to cause injuy to the eyes than Roman letters.

